# Incorporating Differential Gene Expression Analysis with Predictive Biomarkers to Identify Novel Therapeutic Drugs for Fuchs Endothelial Corneal Dystrophy

**DOI:** 10.1155/2021/5580595

**Published:** 2021-06-28

**Authors:** Huaming Wen, Ryan A. Gallo, Xiaosheng Huang, Jiamin Cai, Shaoyi Mei, Ammad Ahmad Farooqi, Jun Zhao, Wensi Tao

**Affiliations:** ^1^Department of Ophthalmology, Shenzhen People's Hospital (The Second Clinical Medical College, Jinan University; The First Affiliated Hospital, Southern University of Science and Technology), Shenzhen 518020, Guangdong, China; ^2^Department of Ophthalmology, Baoan Central Hospital of Shenzhen, The Fifth Affiliated Hospital of Shenzhen University, Shenzhen, Guangdong 518102, China; ^3^Dr. Nasser Al-Rashid Orbital Vision Research Center, Bascom Palmer Eye Institute, Department of Ophthalmology, University of Miami Miller School of Medicine, Miami, FL, USA; ^4^School of Ophthalmology & Optometry Affiliated to Shenzhen University, Shenzhen Eye Hospital, Shenzhen 518040, Guangdong, China; ^5^Shenzhen Eye Hospital Affiliated to Jinan University, Shenzhen Eye Institute, Shenzhen 518040, Guangdong, China; ^6^Institute of Biomedical and Genetic Engineering (IBGE), Islamabad 44000, Pakistan; ^7^Department of Radiation Oncology, University of Miami Miller School of Medicine, Miami, FL, USA

## Abstract

**Purpose:**

Based on the differential gene expression analysis for predictive biomarkers with RNA-Sequencing data from Fuchs endothelial corneal dystrophy (FECD) patients, we are aiming to evaluate the efficacy of Library of Integrated Network-based Cellular Signatures (LINCS) perturbagen prediction software to identify novel pharmacotherapeutic targets that can revert the pathogenic gene expression signatures and reverse disease phenotype in FECD.

**Methods:**

A publicly available RNA-seq dataset was used to compare corneal endothelial specimens from controls and patients with FECD. Based on the differential gene expression analysis for predictive biomarkers, we evaluated the efficacy of LINCS perturbagen prediction software to identify novel therapeutic targets that can revert the pathogenic gene expression signatures and reverse disease phenotypes in FECD.

**Results:**

The RNA-seq dataset of the corneal endothelial cells from FECD patients revealed the differential gene expression signatures of FECD. Many of the differential expressed genes are related to canonical pathways of the FECD pathogenesis, such as extracellular matrix reorganization and immunological response. The expression levels of genes *VSIG*2, *IL*18, and *ITGB*8 were significantly increased in FECD compared with control. Meanwhile, the expression levels of *CNGA*3, *SMOX*, and *CERS*1 were significantly lower in the FECD than in control. We employed LINCS L1000 Characteristic Direction Signature Search Engine (L1000-CDS2) to investigate pathway-based molecular treatment. L1000-CDS2 predicted that small molecule drugs such as histone deacetylase (HDAC) inhibitors might be a potential candidate to reverse the pathological gene expression signature in FECD.

**Conclusions:**

Based on differential gene expression signatures, several candidate drugs have been identified to reverse the disease phenotypes in FECD. Gene expression signature with LINCS small molecule prediction software can discover novel preclinical drug candidates for FECD.

## 1. Introduction

Fuchs endothelial corneal dystrophy (FECD) is a rare corneal genetic disease that can cause vision problems such as blurred vision, corneal opacification, and lower visual acuity [1]. In the United States, FECD dystrophy is a common eye condition affecting about 4 percent of people over the age of 40 [2, 3]. FECD-associated genetic alterations can cause the dysfunction or loss of corneal endothelial cells [4, 5]. FECD is the leading cause of corneal endothelial transplants around the world [6]. There is evidence that FECD is more common in Europe than in other parts of the world and that a higher proportion of patients in Europe and the United States undergo transplants as a result of FECD [1]. Meanwhile, epidemiological data suggested that Asian countries have lower FECD rates [7].

Cornea endothelial cells are a monolayer of hexagonal cells that lay in the back of cornea. They are a layer of cells responsible for the maintenance of corneal stromal dehydration and transparency [4]. Cornea endothelial cells regulate the fluid inside cornea by constantly pumping ions. In FECD, cornea endothelial cells undergo premature senescence and apoptosis [8].

FECD is a progressive disease that can be clinically defined into several stages. During FECD disease development, altered or loss of endothelial cell function causes excessive fluid to accumulate inside stroma of the cornea, a condition named corneal edema [9]. FECD usually begins with focal deposit named guttae in the center of cornea and eventually spreads to the whole cornea [10, 11]. Guttae are associated with cell death within the endothelial cells layer, cornea edema, and vision loss [11]. FECD usually progresses slowly from central to peripheral and from focal to the whole cornea, eventually leading to severe corneal damage and corneal blindness.

The medical management for FECD patients was traditionally performed with ointments or hypertonic saline [9]. Early-stage FECD patients are treated with prescription eye drops or ointments with sodium chloride to reduce pain and swelling [3, 12, 13]. However, when the disease progresses and corneal decompensation starts, surgical treatment becomes necessary. Significant corneal damage may warrant a corneal transplant. Keratoplasty surgery is usually needed to transplant the inner layer of cornea by Descemet membrane endothelial keratoplasty (DMEK) or Descemet-stripping endothelial keratoplasty (DSEK) [14].

Until now, there has been no pharmaceutical therapy approved by the US Food and Drug Administration for FECD, a variety of new drug candidates investigated in this study that could provide new therapeutic options for managing Fuchs endothelial corneal dystrophy patients in the future. Understanding how the disease develops, nonsurgical therapy targeting cell signaling pathway underlying the disease progression may be a promising strategy.

Gene expression analysis using microarray or RNA-sequencing revealed the unique gene expression profile with differentially expressed genes comparing Fuchs endothelial corneal dystrophy patients with controls. Several studies using gene expression analysis have revealed the molecular mechanism of FECD disease [15, 16]. We speculate that these unique gene expression signatures can contribute to elements of underlying disease pathogenesis and progression, such as upregulation in inflammation and downregulation in glycolysis. Therefore, investigating pathway-based molecular treatment opportunities that can effectively target predictive biomarkers and selectively reverse differential gene expression in corneal cells could be a promising therapeutic methodology.

## 2. Materials and Methods

### 2.1. Data Source and Data Normalization

Raw RNA-seq data for the GEO dataset (GSE101872) was downloaded from the SRA database (https://www.ncbi.nlm.nih.gov/geo/query/acc.cgi?acc=GSE101872) and quantified with the ARCHS4 pipeline to gene-level counts [17]. From the ARCHS4 gene expression matrix v5, gene counts were downloaded. By dividing each column by the total number of its counts, multiplying it by 106, followed by the application of a log10-transform, raw counts were normalized to log10-Counts Per Million (logCPM).

### 2.2. PCA and Signature Generation

Principal Component Analysis was performed using the PCA function from the sklearn Python module. Prior to performing PCA, the raw gene counts were normalized using the logCPM method, filtered by selecting the 2500 genes with most variable expression, and finally transformed using the Z-score method. By comparing gene expression levels between the control group and the experimental group using the limma R kit, the gene expression signature was created [18]. Using the methods mentioned for the differential gene expression, the gene expression signature was created by performing differential gene expression analysis.

### 2.3. Clustergrammer

Clustergrammer was used to produce the heatmap [19]. The raw gene counts were normalized using the logCPM method prior to displaying the heatmap, filtered by selecting the 2500 genes with the most variable expression, and finally transformed using the Z-score method.

### 2.4. Volcano Plot

Gene fold changes were transformed using log2 and displayed on the *x*-axis; *P* values were corrected using the Benjamini-Hochberg method, transformed using log10, and displayed on the *y*-axis. See the differential gene expression section for more information on the methods used to generate these values.

### 2.5. Gene Ontology Enrichment Analysis

By analyzing the upregulated and downregulated gene sets using Enrichr, enrichment results were generated with GO Biological Process 2018 [20]. The following libraries were used for the study. Using a cut-off of *P* value <0.1 after applying the Benjamini-Hochberg correction, important terms are calculated.

### 2.6. Pathway Enrichment Analysis

By analyzing the upregulated and downregulated gene sets using Enrichr, enrichment results were generated [20]. For the study, the following libraries were used: KEGG 2016, Reactome 2016, and WikiPathways 2016. Using a cut-off of *P* value <0.1 after applying the Benjamini-Hochberg correction, important terms are calculated.

### 2.7. Transcription Factor Enrichment Analysis

Proteins involved in the transcriptional regulation of gene expression are transcription factors (TFs). A large number of associations between TFs and their transcriptional targets are found in databases such as ChEA and ENCODE. Enrichr uses this data to classify the transcription factors whose targets are overrepresented in the upregulated and downregulated genes found by comparing two groups of samples.

### 2.8. L1000CDS^2^ Query

L1000CDS^2^ is a web-based tool for querying gene expression signatures against signatures created from human cell lines treated with over 20,000 small molecules and drugs for the LINCS project. The L1000CDS^2^ analysis [21] was performed by submitting to the L1000CDS^2^ signature search API the top 2000 genes in the gene expression signature. See the differential gene expression section for more details on the techniques used to produce the signature.

## 3. Results

RNA-seq data from the corneal endothelia of Fuchs endothelial corneal dystrophy patients and controls revealed several differential expressed pathways. The GEO dataset GSE101872 is loaded and analyzed by BioJupies. Expression data was quantified as gene-level counts using the ARCHS4 pipeline [17]. Sample metadata associated with the samples in the RNA-seq dataset are displayed in Table 1. Rows represent RNA-seq samples, and columns represent metadata categories: There are two control samples and 5 Fuchs endothelial corneal dystrophy cases with expansion.

**Figure 1:**
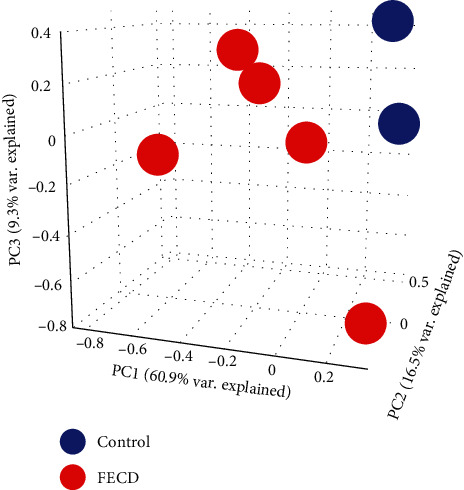
Principal Component Analysis of RNA-seq data from FECD patients. The 3-dimensional figure displays a scatter plot of the first three Principal Components (PCs) of the data. Each point represents an RNA-seq sample. Samples are clustered in the three-dimensional space based on their similar gene expression profile.

BioJupies is a web-based Jupyter notebook (Python developer) integrated with several components such as Principal Component Analysis (PCA), clusters, volcano plots, and Gene Ontology. PCA is commonly used to explore the similarity of biological samples in RNA-seq datasets. To achieve this, gene expression values are transformed into Principal Components (PCs), a set of linearly uncorrelated features which represent the most relevant sources of variance in the data, and subsequently visualized using a 3-dimensional scatter plot. In Figure 1, the control and perturbation groups (FECD) are indicated using different colors. Control groups and FECD groups are distinctively segregated. Clustergrammer is a web-based tool for visualizing and analyzing high-dimensional data as interactive and hierarchically clustered heatmaps. It is used to explore the similarity between samples in an RNA-seq dataset. From the clustergrammer, we can see the different degree of disease progression of FECD from normal to pathological state.

**Figure 2:**
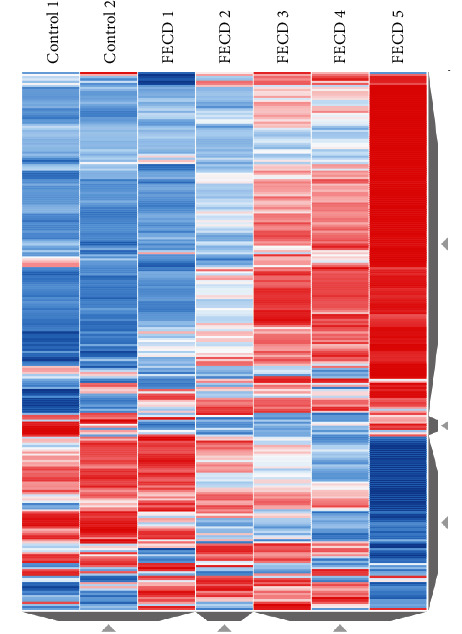
Heatmap visualization of RNA-seq data from FECD patients. For each sample in the RNA-seq dataset, the figure includes a heatmap demonstrating gene expression. Each row of the heatmap represents a gene, each column represents a sample, and each cell displays normalized gene expression values. Blue color represents low expressed genes and red color represents highly expressed genes.

In addition to identifying clusters of samples, it also allows to identify the genes that contribute to the clustering. In Figure 2, we can see the pattern of progression of disease from control to FECD with expansion. In this figure, gene expression signatures were quantified using differential gene expression (DGE) methods, which compare gene expression levels between two groups of samples to identify significantly altered genes in FECD. The signature table is used to interactively display the results of such analyses. Differential expression table (Supplementary Table 1) of RNA-seq disclosed several differentially expressed genes when comparing FECD and controls, including genes such as upregulated genes *VSIG*2*, MATN*2, *TSPAN*13, *L*3*MBTL*3, *ITGB*8, and *BST*2 and downregulated genes *TMEM*132*C, C*1*QL*1*, KCNJ*10, *NEFL, KDM*3*A, PCSK*2, *HILPDA, SEPALLATA*3, and *NES.*

**Figure 3:**
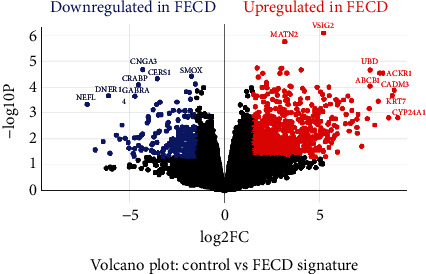
Volcano plot display of differentially expressed genes. A scatter plot showing the log2-fold modifications and statistical significance of each gene determined by conducting a differential analysis of gene expression is included in the figure. Every point in the plot represents a gene. Red points indicate genes that are upregulated, blue points indicate genes that are downregulated.

Volcano plots can be used to quickly identify genes whose expression is significantly altered in a perturbation and to assess the global similarity of gene expressions in control and FECD samples. Each point in the scatter plot represents a gene; the axes display the significance versus fold change estimated by the differential expression analysis. From our analysis in the volcano plot (Figure 3), we found that the genes upregulated in FECD are *MATN*2*, VSIG*2*, UBD, ABCB*1*, ACKR*1 *CADM*3*, KRT*3, and *CYP*24*A*1, and the downregulated genes in FECD are *CNGA*3, *SMOX, CERS*1*, CRABP*1, *GABRA*4*, DNER*, and *NEFL.*

**Figure 4:**
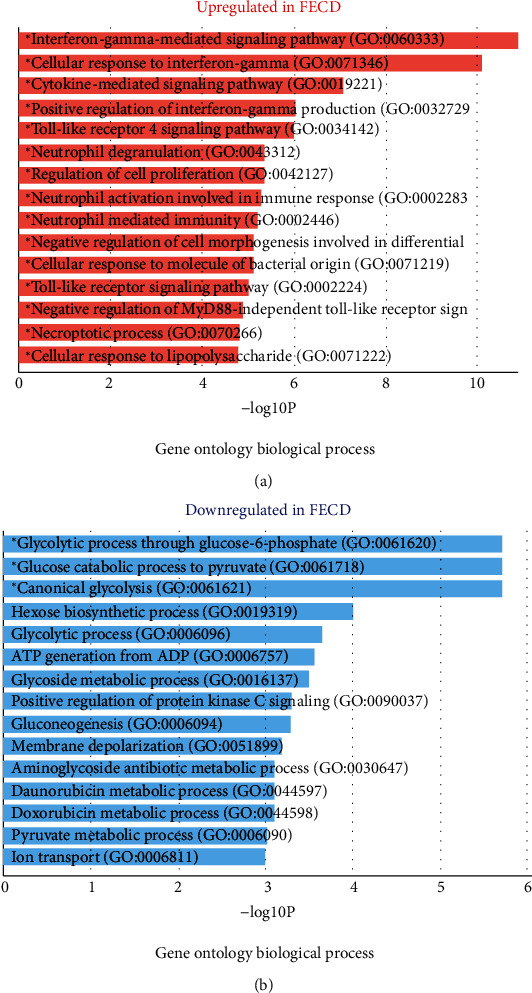
Gene Ontology (GO) enrichment analysis of genes within biological categories. The figure contains bar charts showing the results of the enrichment analysis of Gene Ontology developed using Enrichr. For each term, the *x*-axis indicates the −log10 (*P* value).

Gene Ontology (GO) contains a large collection of experimentally validated and predicted associations between genes and biological terms. This information can be leveraged by Enrichr to identify the biological processes, molecular functions, and cellular components which are overrepresented in the upregulated and downregulated genes identified by comparing two groups of samples, control and FECD patients. In Figure 4, the Gene Ontology biological process upregulated in FECD is as follows: (1) interferon-gamma-mediated signaling pathway (GO-00603330), (2) cellular response to interferon-gamma (GO:0071346), (3) cytokine-mediated signaling pathway (GO:0019221), (4) positive regulation of interferon-gamma production, and (5) Toll-like receptor 4 signaling pathway (GO:0034142). All these pathways are related to immune response such as Toll-like receptor 4 (*TLR*4) and interferon-gamma (*IFN-r*). However, we have the following: (1) the Gene Ontology biological process downregulated in FECD which is a glycolytic process through glucose-6-phosphate (GO:0061620), (2) glucose catabolic process to pyruvate (GO:0061718), and (3) canonical glycolysis (GO:0061621). All these pathways are related to glycolysis.

**Figure 5:**
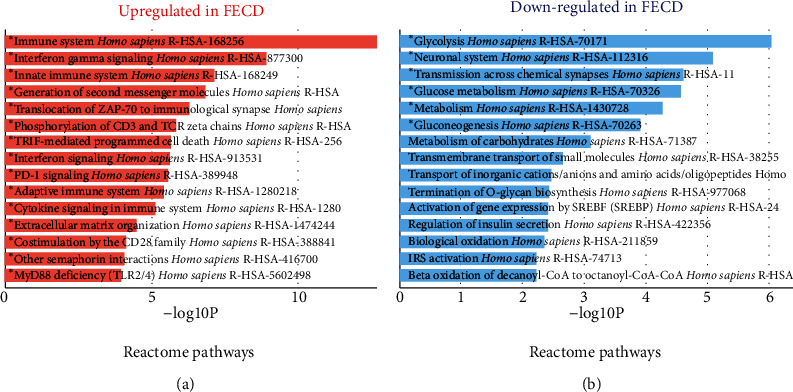
Pathway enrichment analysis identifying significantly impacted pathways. The enrichment results are now displayed as a summary of enriched terms displayed as bar generated using Enrichr. For each term, the *x*-axis indicates the −log10 (*P* value). Significant terms in bold are highlighted.

Biological pathways databases such as KEGG, Reactome, and WikiPathways contain many associations between such pathways and genes. This information can be leveraged by Enrichr to identify the biological pathways which are overrepresented in the upregulated and downregulated genes identified by comparing two groups of samples. In Figure 5, the Reactome pathways upregulated in FECD are (1) immune system signaling (168256), (2) interferon-gamma signaling (877300), and (3) Innate Immune System (168249). Meanwhile, the reactome pathways downregulated in FECD are (1) the reactions of glycolysis (70171), (2) neuronal system pathway (112316), and (3) transmission across chemical synapses (112315). These pathways indicated that FECD is associated with decrease in glycolysis, loss of neuron innervation, and synaptic activities.

Transcription factors (TFs) databases such as ChEA and ENCODE contain a large number of associations between TFs and their transcriptional targets. This information can be leveraged by Enrichr to identify the transcription factors whose targets are overrepresented in the upregulated and downregulated genes identified by comparing two groups of samples from controls and FECD (Supplementary Table 2). Upregulated TFs are EZH2, RNF2, NR1H3, RELA, and RNF166, and downregulated TFs are SOHLH1, OLIG1, RAPGEF5, DEAF1, and ZIC4.

Protein kinases are enzymes that modify other proteins by chemically adding phosphate groups. Databases such as KEA contain a large number of associations between kinases and their substrates. This information can be leveraged by Enrichr to identify the protein kinases whose substrates are overrepresented in the upregulated and downregulated genes identified by comparing two groups of samples from controls and FECD patients (Supplementary Table 3). Upregulated kinases are CSF1R, MAP3K8, RIPK2, MKNK1, and SYK, and downregulated kinases are PNCK, LMTK3, TYRO3, DCLK2, and MAST1.

**Figure 6:**
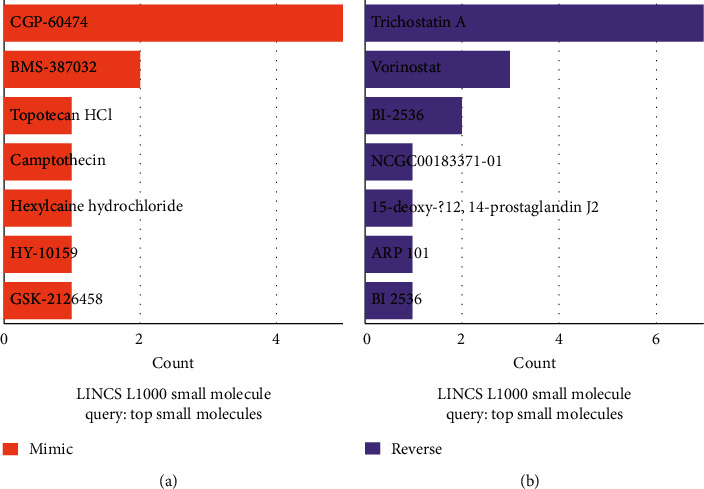
L1000CDS^2^ identify drug candidates that reverse the differential expression signatures. A bar chart showing the top small molecules found by the L1000CDS^2^ query is contained in the figure. The left panel shows the small molecules that imitate the signature of gene expression observed, while the small molecules that reverse it are seen on the right panel.

We explored the database L1000CDS^2^ to find drugs that can reverse disease phenotypes. L1000CDS^2^ is a web-based tool for querying gene expression signatures against signatures created from human cell lines treated with over 20,000 small molecules and drugs for the LINCS project. It is commonly used to identify small molecules which mimic or reverse the effects of a gene expression signature generated from a differential gene expression analysis. In Figure 6, the reverse signature query included trichostatin A, vorinostat, and BI 2536. Trichostatin A and vorinostat are histone deacetylase (HDAC) inhibitors. BI-2536 is a selective inhibitor of Plk1, which inhibits Plk1 enzyme activity.

## 4. Discussion

Corneas are generally considered as an “immune privileged” site, which are able to tolerate foreign antigens without causing an inflammatory immune response [22]. In the clinics, a cornea transplant takes advantage of this unique property. However, in FECD patients, it is well known that immune response plays an important role in disease progression [16, 23]. Our RNA-seq analysis has confirmed previous finding that there is significant upregulation of pathways related to immune response, such as Toll-like receptor 4 (TLR4) and interferon-gamma (IFN-g) [16]. Similar findings of immune response gene changes in our RNA-seq analysis suggested that the methodology we employed can reproduce the findings in the previous literature.

From our RNA-seq analysis, we found that pathways related to glycolysis and neuronal system pathway were downregulated. Loss of glycolysis can be partially explained by the decrease of general metabolic activities. The corneal endothelium cells are responsible for maintaining the homeostasis of cornea by pumping out water osmotically, from the corneal stroma into the aqueous humor [24]. In the corneal endothelial cells, the metabolic pathways of glycolysis and subsequent oxidative phosphorylation are the source of the adenosine triphosphate (ATP), which is necessary to maintain the normal function of the fluid pump [25]. In the case of FECD cornea, loss of endothelial cells and edema in the corneal stroma can be the metabolic consequence of decrease of glycolysis pathway. Meanwhile, due to the sensory function, cornea is densely innervated with nerve fibers via the ophthalmic division of the trigeminal nerve [26]. These nerves form the corneal subbasal nerve plexus which is visible in the central cornea of healthy subjects [23, 27]. However, in the FECD patients, there are alterations in corneal nerve morphology and function in different disease stages. Increasing severity of FECD is correlated with attenuation of the density as well as diminishment of the neuronal function of the subbasal corneal nerve [23, 26, 27]. Our finding in the RNA-seq, indicating the dysregulation of pathways related to neuronal system and transmission across chemical synapses, is consistent with loss of innervation in FECD cornea.

To find new interventions for FECD, we have several strategies to reverse disease phenotypes. Firstly, we can either inhibit the immune responses such as Toll-like receptor 4 (TLR4) and interferon-gamma (IFN-g) [16]. Secondly, we can target the transcription factors whose targets are overrepresented in the upregulated genes in FECD, including *EZH*2, *RNF*2, *NR*1*H*3, *RELA*, and *RNF*166. At the same time, we can also target the protein kinases whose substrates are overrepresented in the upregulated genes in FECD. These upregulated kinases are CSF1R, MAP3K8, RIPK2, MKNK1, and SYK. Lastly, we found several candidate drugs that can reverse disease phenotypes. These drugs include histone deacetylase (HDAC) inhibitors and Plk1 inhibitors. HDAC inhibitors (HDACi) are potential immunomodulators, since they can regulate the production of cytokine, such as L-6, IL-12, and IFN-*γ*, and affect the immune surveillance [28]. HDACi can alter the function and activity of dendritic cell and macrophage and regulate the gene transcriptional activities of interferon-stimulating genes [29]; HDACi can modulate the effector cells of both innate and adaptive immune system. All these strategies can shed light on future pharmacological intervention for FECD.

## Figures and Tables

**Table 1:** Sample metadata.

Sample_geo_accession	Sample title	Subject status	Tissue
GSM2717439	2011–020 (FECD with expansion)	Fuchs endothelial corneal dystrophy	Corneal endothelium
GSM2717440	2011–024 (FECD with expansion)	Fuchs endothelial corneal dystrophy	Corneal endothelium
GSM2717441	2011–038 (FECD with expansion)	Fuchs endothelial corneal dystrophy	Corneal endothelium
GSM2717442	2011–041 (FECD with expansion)	Fuchs endothelial corneal dystrophy	Corneal endothelium
GSM2717443	6004 (FECD with expansion)	Fuchs endothelial corneal dystrophy	Corneal endothelium
GSM2717444	Control 1	Control	Corneal endothelium
GSM2717445	Control 2	Control	Corneal endothelium

The table displays the metadata associated with the samples in the RNA-seq dataset. Rows represent RNA-seq samples, and columns represent metadata categories.

## Data Availability

Raw RNA-seq data for GEO dataset GSE101872 were downloaded from the SRA database (https://www.ncbi.nlm.nih.gov/geo/query/acc.cgi?acc=GSE101872).

## Supplementary Materials

Supplementary Materials Supplementary Table 1: differential expression table. The figure displays a browsable table containing the gene expression signature generated from a differential gene expression analysis. Every row of the table represents a gene; the columns display the estimated measures of differential expression. Supplementary Table 2: transcription factor enrichment analysis results. The table contains scrollable tables displaying the results of the transcription factor (TF) enrichment analysis generated using ENCODE libraries, indicating TFs whose experimentally validated targets are enriched. Supplementary Table 3: kinase enrichment analysis results. The figure contains browsable tables displaying the results of the protein kinase (PK) enrichment analysis generated using the ARCHS4 library, indicating PKs whose top coexpressed genes (according to the ARCHS4 dataset) are enriched.
