# Supplementary material for: Incorporating Differential Gene Expression Analysis with Predictive Biomarkers to Identify Novel Therapeutic Drugs for Fuchs Endothelial Corneal Dystrophy
Source: J Ophthalmol. 2021 Jun 28;2021:5580595. doi: 10.1155/2021/5580595 (PMC8260298; doi:10.1155/2021/5580595)
Supplement: Supplementary Materials — Supplementary Table 1: differential expression table. The figure displays a browsable table containing the gene expression signature generated from a differential gene expression analysis. Every row of the table represents a gene; the columns display the estimated measures of differential expression. Supplementary Table 2: transcription factor enrichment analysis results. The table contains scrollable tables displaying the results of the transcription factor (TF) enrichment analysis generated using ENCODE libraries, indicating TFs whose experimentally validated targets are enriched. Supplementary Table 3: kinase enrichment analysis results. The figure contains browsable tables displaying the results of the protein kinase (PK) enrichment analysis generated using the ARCHS4 library, indicating PKs whose top coexpressed genes (according to the ARCHS4 dataset) are enriched. [file 5580595.f1.docx]

**Supplemental materials:**

**Supplemental Table 1:** Differential expression gene of RNA-seq

| gene_symbol | logFC | AveExpr | t | P.Value | adj.P.Val | B |
| --- | --- | --- | --- | --- | --- | --- |
| VSIG2 | 5.22898241 | 1.069327941 | 13.98949311 | 0.00000082 | 0.015008217 | 5.351603003 |
| MATN2 | 3.177317229 | 4.854566391 | 12.62747096 | 0.00000177 | 0.016215324 | 5.812077522 |
| TSPAN13 | 1.722259094 | 4.421022313 | 9.201192489 | 0.0000183 | 0.042120527 | 3.542679716 |
| L3MBTL3 | 3.023991794 | 3.702277792 | 9.176413986 | 0.0000186 | 0.042120527 | 3.529787554 |
| ITGB8 | 2.995211965 | 3.787256653 | 9.127456443 | 0.0000194 | 0.042120527 | 3.492558896 |
| BST2 | 4.039194109 | 1.575010035 | 8.986338919 | 0.0000217 | 0.042120527 | 3.173879336 |
| UBD | 7.677437719 | -1.372613799 | 8.970512796 | 0.0000219 | 0.042120527 | 2.211046763 |
| FN1 | 8.185108447 | 8.683731274 | 8.619641691 | 0.0000292 | 0.042120527 | 3.090527179 |
| ZNF311 | 3.287437948 | -0.241197755 | 8.616181994 | 0.0000293 | 0.042120527 | 2.537038146 |
| ACKR1 | 8.364934303 | 2.539784691 | 8.61223606 | 0.0000294 | 0.042120527 | 3.037249095 |
| AK5 | 2.927022907 | 2.323668147 | 8.591742295 | 0.0000299 | 0.042120527 | 2.999853296 |
| DNAJC22 | 2.026421899 | 2.538975657 | 8.590995899 | 0.0000299 | 0.042120527 | 3.02106636 |
| NREP | 1.789949831 | 5.365682957 | 8.209760334 | 0.0000413 | 0.050427027 | 2.698889442 |
| ARHGAP18 | 2.225511513 | 4.626205367 | 7.696010394 | 0.0000651 | 0.066156192 | 2.245788185 |
| BIN1 | 2.213856792 | 3.524066067 | 7.450348282 | 0.0000817 | 0.066156192 | 2.073147401 |
| PPP2R2B | 3.460116737 | 2.618724314 | 7.329987468 | 0.0000914 | 0.066156192 | 1.979510428 |
| ABCB1 | 7.673149039 | -0.321976707 | 7.325667504 | 0.0000918 | 0.066156192 | 1.664758614 |
| C12ORF56 | 3.502460233 | -1.425320544 | 7.283012719 | 0.0000956 | 0.066156192 | 1.278701585 |
| TMEM26 | 5.509679157 | -2.948563119 | 7.24988349 | 0.0000987 | 0.066156192 | 0.565779488 |
| RP11-119F19.4 | 4.887065395 | -3.418247825 | 7.229741996 | 0.000100582 | 0.066156192 | 0.307713321 |
| IL18 | 1.988020069 | 2.284017449 | 7.222438399 | 0.000101286 | 0.066156192 | 1.874093835 |
| CDC42EP1 | 1.428327415 | 5.18524285 | 7.179354531 | 0.000105553 | 0.066156192 | 1.730141535 |
| PRDM6 | 2.244176926 | 3.26195091 | 7.152762926 | 0.000108287 | 0.066156192 | 1.804315272 |
| CPVL | 4.11933814 | 4.014452994 | 7.117334639 | 0.000112052 | 0.066156192 | 1.721164904 |
| CNTN3 | 2.850346029 | 4.190767774 | 7.103767526 | 0.000113532 | 0.066156192 | 1.695767401 |
| CNGA3 | -4.305298652 | 0.488537082 | -9.007052074 | 0.0000213 | 0.042120527 | 2.972362476 |
| SMOX | -1.735429473 | 3.520666226 | -8.306891093 | 0.000038 | 0.049702362 | 2.832552146 |
| CERS1 | -3.529372789 | -1.300174079 | -8.061137935 | 0.000047 | 0.053800047 | 1.79027687 |
| NRIP3 | -1.537190036 | 4.190122251 | -7.517239313 | 0.0000767 | 0.066156192 | 2.09767691 |
| CRABP1 | -4.535117635 | 0.091708968 | -7.457302553 | 0.0000811 | 0.066156192 | 1.850176538 |
| FKBP5 | -1.101712986 | 6.029946831 | -7.185405383 | 0.000104942 | 0.066156192 | 1.741420922 |
| TPCN1 | -1.442158334 | 6.104898641 | -6.886997737 | 0.000140381 | 0.066156192 | 1.440337229 |
| PFKFB4 | -2.005579464 | 4.676206956 | -6.8151915 | 0.000150774 | 0.066156192 | 1.374689196 |
| SLC7A14 | -1.850590129 | 2.220350401 | -6.753473776 | 0.000160392 | 0.06829356 | 1.437696474 |
| SCARB1 | -1.211004705 | 6.709235389 | -6.71412793 | 0.000166878 | 0.069201202 | 1.278449465 |
| DNER | -6.115084742 | 2.419702745 | -6.456041839 | 0.00021737 | 0.07119516 | 1.145427125 |
| GABRA4 | -4.713799507 | -1.147071205 | -6.412894763 | 0.00022736 | 0.07119516 | 0.760263006 |
| S1PR3 | -1.467006009 | 3.849806889 | -6.282251882 | 0.000260844 | 0.077838069 | 0.86577334 |
| STX1B | -1.799444855 | 3.003544196 | -6.171090676 | 0.00029366 | 0.081053928 | 0.822653894 |
| TMEM132C | -2.497772527 | -0.460044843 | -5.932155665 | 0.000380788 | 0.08922248 | 0.493839452 |
| C1QL1 | -3.389696576 | 2.796830555 | -5.920245412 | 0.000385824 | 0.08922248 | 0.564091817 |
| KCNJ10 | -3.571248533 | 1.189102896 | -5.747813116 | 0.000467593 | 0.097787962 | 0.415082288 |
| NEFL | -7.238456347 | -3.307562407 | -5.746507807 | 0.000468281 | 0.097787962 | -0.431042197 |
| KDM3A | -1.303746395 | 6.023942065 | -5.467837688 | 0.00064416 | 0.121586768 | -0.159915848 |
| PCSK2 | -3.749895397 | -0.222843953 | -5.333626962 | 0.000753878 | 0.129507942 | -0.067357967 |
| HILPDA | -1.39418218 | 2.843321796 | -5.296693452 | 0.000787564 | 0.130969533 | -0.157427287 |
| SEPALLATA3 | -2.696717322 | 1.768061945 | -5.279208578 | 0.000804085 | 0.130969533 | -0.107890075 |
| NES | -1.812662566 | 4.319672142 | -5.273108132 | 0.000809939 | 0.130969533 | -0.360039993 |
| GRB10 | -2.080684607 | 4.047874197 | -5.245216149 | 0.000837304 | 0.130969533 | -0.367407289 |
| SLC47A1 | -2.380870731 | 2.71118321 | -5.238455857 | 0.000844088 | 0.130969533 | -0.214545612 |

**Supplemental Table 2: Transcription Factor Enrichment Analysis Results**

| term_name | zscore | pvalue | geneset |
| --- | --- | --- | --- |
| EZH2 18974828 ChIP-Seq MESCs Mouse | 1.966205837 | 1.62E-07 | upregulated |
| RNF2 18974828 ChIP-Seq MESCs Mouse | 1.966205837 | 1.62E-07 | upregulated |
| NR1H3 23393188 ChIP-Seq ATHEROSCLEROTIC-FOAM Human | 2.537562604 | 1.62E-07 | upregulated |
| RELA 24523406 ChIP-Seq FIBROSARCOMA Human | 1.996615905 | 3.13E-07 | upregulated |
| SOHLH1 human tf ARCHS4 coexpression | 3.210702341 | 5.75E-07 | downregulated |
| RNF166 human tf ARCHS4 coexpression | 3.210702341 | 5.75E-07 | upregulated |
| OLIG1 human tf ARCHS4 coexpression | 3.210702341 | 5.75E-07 | downregulated |
| RAPGEF5 human tf ARCHS4 coexpression | 3.210702341 | 5.75E-07 | downregulated |
| DEAF1 human tf ARCHS4 coexpression | 3.210702341 | 5.75E-07 | downregulated |
| ZIC4 human tf ARCHS4 coexpression | 3.210702341 | 5.75E-07 | downregulated |

| term_name | zscore | pvalue | geneset |
| --- | --- | --- | --- |
| CSF1R human kinase ARCHS4 coexpression | 7.62541806 | 9.30E-34 | upregulated |
| MAP3K8 human kinase ARCHS4 coexpression | 6.688963211 | 6.39E-27 | upregulated |
| RIPK2 human kinase ARCHS4 coexpression | 6.555183946 | 5.47E-26 | upregulated |
| MKNK1 human kinase ARCHS4 coexpression | 6.02006689 | 2.26E-22 | upregulated |
| SYK human kinase ARCHS4 coexpression | 6.02006689 | 2.26E-22 | upregulated |
| MERTK human kinase ARCHS4 coexpression | 5.752508361 | 1.23E-20 | upregulated |
| CAMK1 human kinase ARCHS4 coexpression | 5.752508361 | 1.23E-20 | upregulated |
| LYN human kinase ARCHS4 coexpression | 5.484949833 | 5.95E-19 | upregulated |
| BTK human kinase ARCHS4 coexpression | 5.484949833 | 5.95E-19 | upregulated |
| PNCK human kinase ARCHS4 coexpression | 4.280936455 | 4.83E-12 | downregulated |
| LMTK3 human kinase ARCHS4 coexpression | 4.147157191 | 2.39E-11 | downregulated |
| TYRO3 human kinase ARCHS4 coexpression | 4.147157191 | 2.39E-11 | downregulated |
| DCLK2 human kinase ARCHS4 coexpression | 4.147157191 | 2.39E-11 | downregulated |
| MAST1 human kinase ARCHS4 coexpression | 4.147157191 | 2.39E-11 | downregulated |
| BRSK1 human kinase ARCHS4 coexpression | 4.013377926 | 1.14E-10 | downregulated |
| MAPK11 human kinase ARCHS4 coexpression | 4.013377926 | 1.14E-10 | downregulated |

**Supplemental Table 3: Kinase Enrichment Analysis Results**
